# Effect of Dietary sugar beet pulp supplementation on growth performance, nutrient digestibility, fecal Microflora, blood profiles and Diarrhea incidence in weaning pigs

**DOI:** 10.1186/s40781-017-0142-8

**Published:** 2017-08-07

**Authors:** C. L. Yan, H. S. Kim, J. S. Hong, J. H. Lee, Y. G. Han, Y. H. Jin, S. W. Son, S. H. Ha, Y. Y. Kim

**Affiliations:** 1 Department of Agricultural Biotechnology, College of Animal Life Sciences, Seoul National University, 1 Gwanak-ro, Gwanak-gu, Seoul, 08826 Republic of Korea; 2 Department of Agricultural College of Yanbian University, Yanji, Jilin 13300 China; 3 PuKyung Pig Farmers Agricultural Cooperative, Gimhae, 50925 Republic of Korea; 4 College of Agriculture and Life Sciences, Seoul National University, Seoul, 08826 South Korea

**Keywords:** Sugar beet pulp, Weaning pig, Growth performance, Blood profiles, Nutrient digestibility

## Abstract

**Background:**

In 2006, the European Union (EU) has decided to forbid use of antibiotics as growth promoters. Although many researches had been conducted about fiber source as alternatives of antibiotics, there are still lack of reports in the literature about the optimum level of sugar beet pulp supplementation, affecting growth performance and nutrient digestibility in weaning pigs. Therefore, different level of sugar beet pulp was added to diets to determine the effects of sugar beet pulp supplementation on growth performance, nutrient digestibility, fecal microflora, blood profile and incidence of diarrhea in weaning pigs.

**Methods:**

A total of 200 weaning pigs [(Yorkshire × Landrace) × Duroc], averaging 9.01 ± 1.389 kg of initial body weight were, allotted to 5 treatments in a randomized complete block (RCB) design. Each treatment was composed of 4 replicates with 10 pigs per pen. The treatments were control treatment: Corn-SBM basal diet + ZnO (phase 1: 0.05%; phase 2; 0.03%) and four different levels of sugar beet pulp were supplemented in Corn-SBM basal diet (3, 6, 9 or 12%). Two phase feeding programs (phase 1: 1–2 weeks; phase 2: 3–5 weeks) were used for 5 week of growth trial.

**Results:**

In feeding trial, there were no significant differences in growth performance and incidence of diarrhea among treatments. The *E.coli* counts were not significantly different among dietary treatments but linear response was observed in *Lactobacillus* counts as sugar beet pulp supplementation increased (*P* < 0.05). In addition, IGF-1, IgA and IgG were not affected by dietary treatments. However, the BUN concentration was decreased when pigs were fed the treatments of diets with SBP compared to that of control treatment (*P* < 0.05). In nutrient digestibility, crude fiber and NDF digestibilities were improved as the sugar beet pulp increased (*P* < 0.05). However, digestibilities of crude ash, crude fat, crude fiber and nitrogen retention were not affected by dietary sugar beet pulp levels.

**Conclusion:**

This experiment demonstrated that sugar beet pulp can be supplemented in weaning pigs’ diet instead of ZnO to prevent postweaning diarrhea without any detrimental effect on growth performance.

## Background

Antibiotics have been used in livestock feeding to increase growth performance and to prevent disease. However, in 2006, the European Union (EU) has decided to forbid use of antibiotics as growth promoters [1]. Also, in Korea, using antibiotics as growth promoters have been banned from 2011. The ban on antibiotics has been reported as the cause of increasing diarrhea followed by impaired growth performance and high mortality of weaning pigs [2].

Feeding a minimum level of fiber can support normal physiological activity in the digestive tract [3]. Diets or ingredients which have high fiber content in young pigs may affect as negatively to voluntary feed intake and nutrient digestibility, respectively [4]. Recent research, it has shown that dietary fiber supplementation can reduce the incidence of diarrhea and improve performance in weaning pigs [5, 6]. Dietary fiber includes soluble dietary fiber (SDF) and insoluble dietary fiber (IDF). Due to supplemented SDF to weaning pigs, it gives the positive effect to decrease the incidence of diarrhea and improve to gut health. Because of high water holding capacity, SDF could affect to those facts [7]. In addition, the bacteria from small and large intestine could affect to degrade the most of SDF and partial of IDF [7–9]. When SDF arrived into the large intestine, the degree of fermentation is faster and better than IDF [10, 11]. In the large intestine, number and activity of microbes are increased by SDF and it also work in the ileum as well [3].

Sugar beet pulp (SBP) includes high level of soluble fiber such as pectins and glucans [12]. However, there are still lack of reports in the literature about the optimum level of sugar beet pulp supplementation, affecting growth performance and nutrient digestibility in weaning pigs. Therefore, present study was conducted to determine the effects of sugar beet pulp supplementation on growth performance, nutrient digestibility, fecal microflora, blood profile and incidence of diarrhea in weaning pigs.

## Methods

### Animal and management

All experimental procedures involving animals were conducted in accordance with the Animal Experimental Guidelines provided by the Seoul National University Institutional Animal Care and Use Committee (SNU-IACUC; SNU-160613-10).

A total of 200 weaning pigs [(Yorkshire × Landrace) × Duroc] with an average body weight of 9.01 ± 1.389 kg, weaned at 25 ± 3 days. The weaning house temperature was maintained at 31 °C, and then gradually fallen to 26 °C at the end of the experiment. During the 5 weeks feeding trial, weaning pigs were allowed ad libitum access to water and diets.

### Experimental design and diet

Experimental pigs were grouped into a randomized complete block (RCB) design in 4 replicates with 10 pigs per pen. Treatments consisted of 4 different levels of sugar beet pulp (3.0, 6.0, 9.0, 12.0%) and one positive control (PC) treatment. Present study was conducted with corn-SBM-barley based diet and two-phase feeding program was used. Phase 1 diet contained 20.56% crude protein and 1.35% lysine for 0–2 weeks. Phase 2 diet contained 18.88% crude protein and 1.15% lysine for 3–5 weeks. All other nutrients of experimental diets were met or slightly exceeded the nutrient requirements [13]. Formulas and composition of the experimental diets were shown in Tables 1 and 2.

**Table 1:** Formula and chemical composition of diets (phase 1)

Ingredients,%	Treatment^a^				
	Con	SBP3	SBP6	SBP9	SBP12
Corn	25.01	21.61	17.63	13.37	9.63
Soy bean meal	33.16	33.05	32.88	32.67	32.51
Wheat	9.70	9.88	10.60	11.65	12.12
Barley	15.00	15.00	15.00	15.00	15.00
Whey powder	4.00	4.00	4.00	4.00	4.00
Lactose	8.00	8.00	8.00	8.00	8.00
Sugar Beet Pulp	0.00	3.00	6.00	9.00	12.00
Soy-oil	1.73	2.15	2.62	3.09	3.55
MDCP	1.36	1.40	1.42	1.45	1.48
Limestone	1.03	0.95	0.89	0.81	0.74
L-Lysine-HCl, 78%	0.29	0.29	0.29	0.29	0.29
DL-methionine, 80%	0.08	0.08	0.08	0.08	0.09
L-threonine, 99%	0.09	0.09	0.09	0.09	0.09
Vit. Mix^b^	0.10	0.10	0.10	0.10	0.10
Min. Mix^c^	0.10	0.10	0.10	0.10	0.10
Salt	0.30	0.30	0.30	0.30	0.30
Zinc oxide	0.05	0.00	0.00	0.00	0.00
Total	100.00	100.00	100.00	100.00	100.00
Chemical composition					
ME^d^, kcal/kg	3265.04	3265.01	3265.01	3265.00	3265.01
Total lysine^d^,%	1.35	1.35	1.35	1.35	1.35
Total methionine^d^,%	0.35	0.35	0.35	0.35	0.35
Total threonine^d^,%	0.86	0.86	0.86	0.86	0.86
Calcium^d^,%	0.80	0.80	0.80	0.80	0.80
Total phosphorus^d^,%	0.65	0.65	0.65	0.65	0.65
Moisture^e^,%	9.67	9.66	9.01	8.66	8.36
Crude Protein^e^,%	20.71	20.64	20.69	20.80	20.62
Crude Ash^e^,%	6.35	6.36	6.50	6.42	6.09
Ether extract^e^,%	3.20	3.99	4.59	4.74	5.74
Crude fiber^e^,%	3.23	3.79	4.29	5.04	5.65
NDF^e^,%	13.38	11.55	13.13	14.22	17.03
ADF^e^,%	4.27	4.26	5.02	6.00	6.50

^a^ PC: corn-SBM based diet + ZnO 0.5%, SBP3: basal diet + SBP 3%, SBP6: basal diet + SBP 6%, SBP9: basal diet + SBP 9%, SBP12: basal diet + SBP 12%

^b^ Provided the following quantities of vitamins per kg of complete diet: Vit A, 8000 IU; Vit D_3_, 1800 IU; Vit. E, 80 IU; Vit. K_3_, 2 mg; Rivoflavin, 7 mg; Calcium pantothenic acid, 25 mg; Niacin, 27 mg; d–Biotin, 200μg; Vit.B_12_, 50μg

^c^ Provided the following quantities of minerals per kg of complete diet: Fe, 150 mg; Cu, 105 mg; Mn, 51 mg; I, 1 mg; Se, 0.3 mg; Zn, 72 mg

^d^ Calculated value

^e^ Analyzed value

**Table 2:** Formula and chemical composition of diets (phase 2)

Ingredients,%	Treatment^a^				
	Con	SBP3	SBP6	SBP9	SBP12
Corn	35.34	31.93	28.44	24.83	20.98
Soy bean meal	28.68	28.55	28.43	28.31	28.16
Wheat	10.40	10.57	10.78	11.09	11.68
Barley	15.00	15.00	15.00	15.00	15.00
Whey powder	2.00	2.00	2.00	2.00	2.00
Lactose	4.00	4.00	4.00	4.00	4.00
Sugar Beet Pulp	0.00	3.00	6.00	9.00	12.00
Soy-oil	1.73	2.16	2.61	3.07	3.53
MDCP	1.16	1.19	1.22	1.25	1.28
Limestone	0.91	0.84	0.76	0.69	0.61
L-Lysine-HCl, 78%	0.19	0.19	0.19	0.19	0.19
DL-methionine, 80%	0.04	0.05	0.05	0.05	0.05
L-threonine, 99%	0.02	0.02	0.02	0.02	0.02
Vit. Mix^b^	0.10	0.10	0.10	0.10	0.10
Min. Mix^c^	0.10	0.10	0.10	0.10	0.10
Salt	0.30	0.30	0.30	0.30	0.30
Zinc oxide	0.03	0.00	0.00	0.00	0.00
Total	100.00	100.00	100.00	100.00	100.00
Chemical composition					
ME^d^, kcal/kg	3265.01	3265.01	3265.00	3265.01	3265.02
Total lysine^d^,%	1.15	1.15	1.15	1.15	1.15
Total methionine^d^,%	0.31	0.31	0.31	0.31	0.31
Total threonine^d^,%	0.74	0.74	0.74	0.74	0.74
Calcium^d^,%	0.70	0.70	0.70	0.70	0.70
Total phosphorus^d^,%	0.65	0.65	0.65	0.65	0.65
Moisture^e^,%	9.50	9.02	8.99	8.76	8.82
Crude Protein^e^,%	19.23	19.22	18.78	18.86	19.04
Crude Ash^e^,%	6.05	6.09	6.26	5.92	6.14
Ether extract^e^,%	3.77	4.33	4.66	4.92	5.46
Crude fiber^e^,%	3.54	4.45	4.92	6.15	6.56
NDF^e^,%	13.29	13.96	15.15	17.70	18.85
ADF^e^,%	4.14	4.68	5.25	6.54	6.98

^a^ PC: corn-SBM based diet + ZnO 0.3%, SBP3: basal diet + SBP 3%, SBP6: basal diet + SBP 6%, SBP9: basal diet + SBP 9%, SBP12: basal diet + SBP 12%

^b^ Provided the following quantities of vitamins per kg of complete diet: Vit A, 8000 IU; Vit D_3_, 1800 IU; Vit. E, 80 IU; Vit. K_3_, 2 mg; Rivoflavin, 7 mg; Calcium pantothenic acid, 25 mg; Niacin, 27 mg; d–Biotin, 200μg; Vit.B_12_, 50μg

^c^ Provided the following quantities of minerals per kg of complete diet: Fe, 150 mg; Cu, 105 mg; Mn, 51 mg; I, 1 mg; Se, 0.3 mg; Zn, 72 mg

^d^ Calculated value

^e^ Analyzed value

### Growth performance

Body weight and feed consumption were recorded at 0, 2, and 5 weeks to calculate average daily gain (ADG), average daily feed intake (ADFI) and gain to feed ratio (G:F ratio).

### Blood profiles

Blood samples were taken from the jugular vein of randomly selected five pigs in each treatment for measuring blood urea nitrogen (BUN), insulin growth like factor-1(IGF-1), immunoglobulin A (IgA) and G (IgG) after pigs were fasten 3 h. Collected blood samples were centrifuged for 15 min at 3000 rpm on 4 °C (Eppendorf centrifuge 5810R, Germany). Be carefully remove the serums to plastic vials and stored at −20 °C. The concentration of BUN and IGF-1 were analyzed using blood analyzer (Ciba-Corning model, Express plus, Ciba Corning Diagnostics Co.) and IgA, IgG were analyzed by ELISA assay (ELISA Starter Accessory Package, Pig IgA and IgG ELISA Quantitation Kit; Bethyl).

### Digestibility trial

According to a completely randomized design (CRD), fifteen weaning pigs (14.42 ± 0.45 kg) were allotted to 5 treatment with 3 replicates. During the digestibility trial, diet was provided twice per day at 7:00 and 19:00 h by three times the maintenance energy requirement (106 kcal of ME/kg of BW^0.75^) and water was provided ad libitum [14]. After 7 days of adaptation period, fecal and urine samples were collected 5 days. To determine the first and last day of collection days, 0.5% of ferric oxide and chromium oxide were added in the first and last experimental diet as selection marker, respectively. Fecal and urine were collected daily and stored −20 °C then fecal samples were dried in air-forced drying oven at 60 °C for 72 h, and ground into 1 mm particles in a Wiley mill for chemical analysis included moisture, protein, fat and ash contents [15]. Total urine was collected daily in a plastic container containing 50 ml of 4 N H_2_SO_4_ to avoid nitrogen evaporation and frozen during the 5 days of collection period for nitrogen retention analysis.

### Diarrhea incidence

During the whole feeding trial period, diarrhea score was recorded once a day (10:00) by counting the number of pigs with diarrhea per pen. The diarrhea score was from 0 (no pigs with diarrhea) to 10 (all pigs with diarrhea).

### Fecal microflora

Fecal *E.coil* and *Lactobacillus* count were measured at 0, 2 and 5 weeks. Samples were collected 1 g of feces and diluted with 9 ml of distilled water. After mixing the solution, it was taken 1 g and diluted with 9 ml of distilled water again. Like this way of dilution, samples was diluted to 1/10^5^ concentration of initial diluted solution. Each diluted solution was smeared in petridish having MacConkey agar (BBL™, BD, USA) and Lactobacilli MRS (Difco™, BD, USA) Broth respectively. There agar plates were incubated at 37 °C for 24 h for *E.coil* and *Lactobacillus* proliferation. After incubation, the number of fecal *E.coil* and *Lactobacillus* were counted.

### Chemical and statistical analysis

Experimental diet and excreta were analyzed for contents of dry matter (procedure 967.03 [15]); ash (procedure 923.03 [15]). The nitrogen content of feces and urine was analyzed by using the Kjeldahl procedure with Kjeltec (KjeltecTM 2200, Foss Tecator, Sweden) and calculating the CP content (Nitrogen × 6.25; procedure 981.10 [15]). Experimental data were analyzed as a randomized complete block (RCB) design using the General Linear Model (GLM) procedure of SAS. In the growth performance data, a pen was considered an experimental unit, while an individual pig was used as the data unit for data on nutrient digestibility, fecal microflora, blood profile, and incidence of diarrhea. The effects of the levels of sugar beet pulp were analyzed while linear and quadratic components were analyzed by orthogonal polynomial contrasts. Differences were determined significant at *P* < 0.05.

## Results and discussion

### Growth performance

The result of body weight (BW), average daily gain (ADG), average daily feed intake (ADFI) and feed efficiency (G:F ratio) are presented in Table 3. During experimental period, there were no significant differences in BW, ADG, ADFI, G:F ratio. Variable results were showed in the literature with the effect of dietary fiber on growth performance in weaning pigs. Most results reported negative effect of fiber inclusion on growth performance of piglet [16, 17]. In contrast, Longland et al. [18] reported no difference in growth performance of pig weaned at 21 days of age that fed diet with 15% sugar beet pulp. And Gill et al. [19] reported no adverse effects on ADG when fed a cereal-based diet containing 0, 15 and 18.5% of sugar beet pulp to 4–8 week old piglets. In other experiment with 6% sugar beet pulp in a wheat based diet, a positive effect on growth performance was observed after weaning pigs [20]. The growth performance in current study showed no negative effect when weaning pigs were fed diet with sugar beet pulp. The result appeared to conflict the held belief that NSP (non-starch polysaccharide) was cannot be fermented and utilized by young pigs [21, 22].

**Table 3:** Effects of sugar beet pulp levels supplementation on growth performance in weaning pigs^a^

Criteria	Treatment^b^					SEM^c^	*P*-value^d^	
	PC	SBP3	SBP6	SBP9	SBP12		Lin.	Quad.
Body weight, kg								
Initial	9.01	9.01	9.01	9.01	9.01	0.300	-	-
2 week	12.83	12.15	12.48	12.63	12.32	0.380	0.68	0.86
5 week	20.79	19.77	21.21	22.67	21.23	0.625	0.16	0.47
ADG, g								
0–2 week	273	229	248	259	235	11.7	0.80	0.83
3–5 week	379	370	393	478	424	18.9	0.20	0.29
0–5 week	337	314	336	390	349	14.8	0.28	0.37
ADFI, g								
0–2 week	352	308	345	332	293	10.2	0.45	0.73
3–5 week	821	761	857	853	747	22.1	0.83	0.99
0–5 week	634	577	653	645	566	16.3	0.76	0.93
G:F ratio								
0–2 week	0.78	0.73	0.73	0.80	0.81	0.025	0.17	0.52
3–5 week	0.57	0.59	0.64	0.71	0.65	0.033	0.42	0.60
0–5 week	0.68	0.68	0.75	0.81	0.72	0.035	0.58	0.69

^a^A total 200 weaning pigs was fed from average initial body 9.01 ± 1.389 kg

^b^PC: corn-SBM based diet + ZnO, SBP3: basal diet + SBP 3%, SBP6: basal diet + SBP 6%, SBP9: basal diet + SBP 9%, SBP12: basal diet + SBP 12%

^c^Standard error of means

^d^Probability values for the effects of SBP3, SBP6, SBP9, SBP12

### Diarrhea incidence

The incidence of diarrhea was showed in Table 4 and there was no significant difference among treatments in whole experimental period. Sugar beet pulp had a high water holding capacity (WHC) and swelling water capacity (SWC), which increased volume of digest, viscosity and water retention [23, 24]. The results were similar to result of Berrocoso et al. [25]. With those observation, during 7 to 10d postweaning period, pigs adjusted to solid feed consumption and it proved that proper amounts of soluble fiber improved the healthy fermentation of undigested nutrients. Because of gastrointestinal tract (GIT) microflora, it helped to ferment soluble fiber sources and the environment of GIT was also improved and stabled, besides the incidence of diarrhea also was reduced [26]. In addition, weaning pigs 33 to 39 d old with 12% sugar beet pulp in their diet made improved digestive functions and this was better result than other weaning pigs fed control diet [20].

**Table 4:** Effects of sugar beet pulp levels supplementation on incidence of diarrhea in weaning pigs

Criteria	Treatment^a^					SEM^b^	*P*-value^c^	
	PC	SBP3	SBP6	SBP9	SBP12		Lin.	Quad.
Diarrhea score^d^								
0–2 week	1.71	1.57	1.36	1.43	1.43	0.125	0.77	0.70
3–5 week	1.14	0.95	0.81	0.71	0.76	0.075	0.33	0.53
0–5 week	1.37	1.20	1.03	1.00	1.03	0.071	0.42	0.51

^a^ PC: corn-SBM based diet + ZnO, SBP3: basal diet + SBP 3%, SBP6: basal diet + SBP 6%, SBP9: basal diet + SBP 9%, SBP12: basal diet + SBP 12%

^b^ Standard error of means

^c^ 0 (No pigs with diarrhea) - 10 (All pigs with diarrhea)

^d^ Probability values for the effects of SBP3, SBP6, SBP9, SBP12

### Fecal micorflora

The effects of sugar beet pulp supplementation on fecal microflora were showed in Table 5. There were linear response on *Lactobacillus* counts as the level of sugar beet pulp increased in whole experimental period (linear, *P* < 0.05). In contrary, in whole experimental period, *E. coli* counts had no significant differences among treatments. Recently, some authors found that the decrease in the enteric *E. coli* after weaning was smaller when fed diet with ZnO [27]. So present study showed that *E. coil* was on the decrease in weaning pigs within PC treatment and treatments of diet with sugar beet pulp, together. These results were the same with previous researches [11, 28, 29]. Prohaska [30] and May et al. [31] reported that fermentation of dietary fiber produced short chain fatty acids (SCFA), which decreased the gut content pH. In an acidic environment, the growth of intestinal bacterial pathogens was restrained by SCFA. Edwards [32] found that feeding diet with dietary fiber on weaning pigs increased intestinal counts of *Lactobacillus* and reduced the incidence of diarrhea. Soluble dietary fiber increased the number and activity of microbes by soluble dietary fiber in the large intestine, and even in the ileum [3]. And a reduction in pH accelerated growth of beneficial bacteria like *Lactobacillus* [33].

**Table 5:** Effects of sugar beet pulp levels supplementation on fecal microflora in weaning pigs^a^

Criteria	Treatment^b^					SEM^c^	*P*-value^d^	
	PC	SBP3	SBP6	SBP9	SBP12		Lin.	Quad.
*E. coli*, cfu/ml								
Initial	5.84	5.84	5.84	5.84	5.84	0.109	-	-
2 week	3.87	4.44	4.22	4.21	4.87	0.131	0.30	0.12
5 week	4.41	4.00	4.17	5.50	4.33	0.214	0.29	0.17
*Lactobacillus*, cfu/ml								
Initial	6.63	6.63	6.63	6.63	6.63	0.053	-	-
2 week	6.08	6.53	6.36	7.17	6.64	0.130	0.04	0.32
5 week	7.81	8.03	7.96	8.04	8.19	0.047	0.02	0.97

^a^ Least squares means for 4 pigs per treatment

^b^ PC: corn-SBM based diet + ZnO, SBP3: basal diet + SBP 3%, SBP6: basal diet + SBP 6%, SBP9: basal diet + SBP 9%, SBP12: basal diet + SBP 12%

^c^ Standard error of means

^d^ Probability values for the effects of SBP3, SBP6, SBP9, SBP12

### Nutrient digestibility

The effects of sugar beet pulp supplementation on nutrient digestibility and nitrogen retention were showed in Table 6. Crude fiber, neutral detergent fiber (NDF) and acid detergent fiber (ADF) digestibility were improved as dietary sugar beet pulp level increased (*P* < 0.05). There was linear response on NDF digestibility as sugar beet pulp level increased (linear, *P* < 0.05) and crude ash, crude fat, crude fiber and ADF tended to improve linearly as sugar beet pulp level increased (linear, *P* < 0.10). However, nitrogen retention was not affected by the supplementation level of sugar beet pulp. The results showed that increasing level of sugar beet pulp played a positive effects in nutrient digestibility. It was the same as the some previous researches. Bindelle et al. [34] found that there was a linear increase in the digestibility when added 0, 10, 20 and 30% levels of sugar beet pulp fed to growing pigs. In addition, Freire et al. [35] represented that increasing DM (dry matter) digestibility when 20% sugar beet pulp was included in a corn-fishmeal diet and Chabeauti et al. [36] reported that ATTD (apparent total tract digestibility) of GE (gross energy) was increased in growing pigs when fed 16% sugar beet pulp. Also, the diets with 2.5 or 5% sugar beet pulp to feed piglet improved ATTP of all nutrients except crude protein [25]. Varel et al. [37] reported celluloytic bacteria like Fibrovacter succinogenes and Ruminococcus flavefaciens inhabited pig’s large intestine. This was the reason for high fiber utilization or supplementation high level of NSP diets. So there was a positive effect in nutrient digestibility when pigs fed diets with a reasonable level of sugar beet pulp.

**Table 6:** Effects of sugar beet pulp levels supplementation on nutrient digestibility in weaning pigs^1^

Criteria	Treatment^2^					SEM^3^	*P*-value^4^	
	PC	SBP3	SBP6	SBP9	SBP12		Lin.	Quad.
Nutrient digestibility,%								
Dry matter	87.08	89.24	87.31	88.15	89.07	0.534	0.49	0.95
Crude protein	85.13	87.55	86.06	85.10	86.25	0.553	0.96	0.69
Crude ash	57.00	71.15	66.92	65.85	71.31	2.013	0.08	0.35
Crude fat	76.09	84.05	79.31	81.34	86.40	1.425	0.07	0.93
Crude fiber	55.07^b^	66.64^ab^	62.92^ab^	74.14^a^	75.84^a^	2.601	0.05	0.49
ADF	44.89^b^	56.89^ab^	53.92^ab^	64.58^a^	66.49^a^	2.741	0.07	0.58
NDF	61.43^c^	68.64^bc^	67.89^bc^	73.61^ab^	76.77^a^	1.681	0.02	0.40
Nitrogen retention, g/d								
N intake	20.16	20.27	19.92	19.66	20.18	0.059	-	-
Fecal N	3.00	2.52	2.78	2.93	2.77	0.110	0.96	0.60
Urinary N	5.74	4.39	7.13	4.50	4.21	0.394	0.21	0.24
N retention^5^	11.42	13.36	10.02	12.22	13.19	0.447	0.37	0.26

^1^ A total 15 weaning pigs was fed from average initial body 14.42 ± 0.45 kg

^2^ PC: corn-SBM based diet + ZnO, SBP3: basal diet + SBP 3%, SBP6: basal diet + SBP 6%, SBP9: basal diet + SBP 9%, SBP12: basal diet + SBP 12%

^3^ Standard error of means

^4^ Probability values for the effects of SBP3, SBP6, SBP9, SBP12

^5^ N retention = N intake - Fecal N - Urinary N

^a,b,c^ Means with different superscripts within the same row significantly differ (*P* < 0.05)

### Blood profiles

The blood urea nitrogen (BUN) and insulin like growth factor-1 (IGF-1) concentration were showed in Table 7. In 5 week, the treatments of diets with sugar beet pulp presented lower BUN concentration than PC treatment (*P* < 0.05). In whole experimental period, pigs fed diets with sugar beet pulp treatments showed the numerically higher IGF-1 concentration than PC treatment. In general, BUN was the indicator for determination of amino acid utilization by pigs and it was directly related to intake of protein and inversely to quality of protein [38, 39]. Hahn et al. [39] found BUN values had negative correlation with ADG and G:F ratio. Therefore, the difference of BUN at 5 week could be explained by the results that the treatments which sugar beet pulp was added got numerically higher results than PC treatment in ADG or G:F ratio until 3–5 weeks and it might improve gut health by increasing the level of sugar beet pulp supplementation.

**Table 7:** Effects of sugar beet pulp levels supplementation on blood profiles in weaning pigs^a^

Criteria	Treatment^b^					SEM^c^	*P*-value^d^	
	PC	SBP3	SBP6	SBP9	SBP12		Lin.	Quad.
IGF-1, ng/dl								
Initial	53.7	53.7	53.7	53.7	53.7	3.21	-	-
2 week	132.8	158.9	134.6	125.7	113.6	8.31	0.13	0.77
5 week	109.3	169.2	158.5	162.2	153.1	8.40	0.60	0.97
BUN, mg/dl								
Initial	6.96	6.96	6.96	6.96	6.96	0.306	-	-
2 week	10.70	11.06	10.58	11.60	9.48	0.423	0.44	0.45
5 week	12.16^a^	10.40^b^	10.42^b^	9.90^b^	10.08^b^	0.272	0.57	0.89

^a^ Least squares means for 5 pigs per treatment

^b^ PC: corn-SBM based diet + ZnO, SBP3: basal diet + SBP 3%, SBP6: basal diet + SBP 6%, SBP9: basal diet + SBP 9%, SBP12: basal diets + SBP 12%

^c^ Standard error of means

^d^ Probability values for the effects of SBP3, SBP6, SBP9, SBP12

IGF-1 was secreted when the growth hormones were stimulated and affected by nutritional status of animal. IGF-1 played an important role such as energy supply for cell growth, regulation of structure, function of cardiovaccular system and born growth [40]. In 5 weeks, pigs fed diets with sugar beet pulp showed numerically higher than PC treatment. Bhutta et al. [41] reported IGF-1 concentration was increased when someone took nutritional supplement. It could be thought of as improving intestine health by the addition of sugar beet pulp and taking a nutritional supplement by fermentation of sugar beet pulp as dietary fiber.

### Immune response

There was an intestinal microbiota in the GIT providing different benefits to the host and it stimulated the immune system [42, 43]. And the immune system was impacted by VFA (volatile fatty acid) like butyrate [44]. However, the result of Table 8 showed no significant difference in (IgA) and (IgG) during the whole experimental period. So, the results demonstrated that supplementation of sugar beet pulp had no effects on IgA and IgG in weaning pigs.

**Table 8:** Effects of sugar beet pulp levels supplementation on IgA and IgG in weaning pigs^a^

Criteria	Treatment^b^					SEM^c^	*P*-value^d^	
	PC	SBP3	SBP6	SBP9	SBP12		Lin.	Quad.
IgA, mg/ml								
Initial	0.12	0.12	0.12	0.12	0.12	0.009	-	-
2 week	0.22	0.20	0.19	0.39	0.29	0.028	0.10	0.46
5 week	0.46	0.52	0.45	0.50	0.45	0.029	0.68	0.93
IgG, mg/ml								
Initial	2.83	2.83	2.83	2.83	2.83	0.052	-	-
2 week	2.25	2.59	2.17	2.51	2.39	0.095	0.77	0.50
5 week	3.04	3.63	3.05	3.72	3.11	0.125	0.49	0.96

^a^ Least squares means for 5 pigs per treatment

^b^ PC: corn-SBM based diet + ZnO, SBP3: basal diet + SBP 3%, SBP6: basal diet + SBP 6%, SBP9: basal diet + SBP 9%, SBP12: basal diet + SBP 12%

^c^ Standard error of means

^d^ Probability values for the effects of SBP3, SBP6, SBP9, SBP12

## Implication

In conclusion, sugar beet pulp as an alternative ZnO could be supplemented in weaning pigs. In growth performance, there was no significant difference among treatments during the experimental period. And pigs fed the treatments of diets with sugar beet pulp showed numerically lower than the treatment of diet with ZnO in incidence of diarrhea. Also, the increased level of sugar beet pulp supplementation, there was an increase in counts of *Lactobacillus*. There were positive effects in nutrient digestibility when pigs fed diets with sugar beet pulp and addition of sugar beet pulp could improve amino acid utilization and take nutritional supplement. And IgA could be increased by the increased level of sugar beet pulp supplementation. Consequently, sugar beet pulp as an alternative ZnO could be supplemented in weaning pigs without any detrimental effect on growth performance.

## Abbreviations

ADF: Acid detergent fiber
ADFI: Average daily feed intake
ADG: Average daily gain
ATTD: Apparent total tract digestibility
BUN: Blood urea nitrogen
BW: Body weight
CP: Crude protein
CRD: Completely randomized design
DF: Dietary fiber
EU: European union
GE: Gross energy
GF: Ratio Gain to feed ratio
GIT: Gastrointestinal tract
IDF: Insoluble dietary fiber
IgA: Immunoglobulin A
IGF-1: Insulin growth like factor-1
IgG: Immunoglobulin G
ME: Metabolizable energy
NDF: Neutral detergent fiber
NSP: Non starch polysaccharide
PC: Positive control
RCBD: Randomized complete block design
SBM: Soy bean meal
SBP: Sugar beet pulp
SCFA: Short chain fatty acid
SDF: Soluble dietary fiber
SWC: Swelling water capacity
VFA: Volatile fatty acid
WHC: Water holding capacity
ZnO: Zinc oxide
